# Trauma across the life course and pathways to healing for older women experiencing homelessness

**DOI:** 10.3389/fgwh.2025.1718879

**Published:** 2025-11-24

**Authors:** Suzanne L. Wenzel

**Affiliations:** Suzanne Dworak-Peck School of Social Work, University of Southern California, Los Angeles, CA, United States

**Keywords:** homelessness, older women, trauma, gender violence, housing

## Abstract

Older women are increasingly experiencing the trauma of homelessness. The precipitants and concomitants of homelessness are also traumas, are multifold, and reflect women's intersectional identities and experiences over their life course. Older women with experiences of homelessness and other traumas require pathways to healing, a life they experience as full and valued. Housing is a necessary and non-negotiable ingredient in addressing the well-being of older women with experiences of homelessness and other traumas. Research indicates that housing alone is not sufficient to achieve healing from homelessness and other traumas; rather, the combination of Permanent Supportive Housing (PSH) and Trauma-Informed Care (TIC) is essential and foundational. Promising approaches to support healing are examined and proposed as future directions to complement the foundation of PSH and TIC. Multiple structural inequities underlying older women's homelessness must also be addressed through policy action. The need for a fundamental shift in how we, as a society, regard and implement public assistance to combat homelessness is discussed in the context of the “Duty-to-Assist” framework.

## 1 Introduction

Decades ago, scholars called out the “bag lady” epithet that had been affixed to older women experiencing homelessness in the 1970s (1). Scholars served as witness to the lack of care and societal scorn directed at these women (2) and documented the women's physical, social, and emotional destitution (1, 3, 4).

Today, homelessness among women, particularly single women, has increased as a proportion of the total population of persons experiencing homelessness (5). The representation of older adults among persons now homeless has also markedly increased (5–7). Older women experiencing homelessness are at the confluence of these two demographic shifts. Whereas in past decades older women enduring homelessness and other trauma had often been met with neglect or worse, our responses going forward must focus on ensuring effective pathways to healing from the harms of myriad traumas. Based on the work of Gonyea and Melekis (8), I define healing in terms of experiencing a “valued life and self” (8 p68).

In developing pathways to healing, we must recognize the traumas – inclusive of gender-violence, sexism, ageism, and racism, for example – in the lives of older, homeless-experienced women. More attention must be devoted to effectively addressing the needs of the underserved population of older women experiencing homelessness (9).

In this Perspective, I provide an overview of trauma experiences to emphasize the severity, complexity, and insidious effects of trauma over the life course, and thus the profound need to facilitate women's healing. I offer insights on effective, supportive resources drawing from evidence-based research and suggest future directions to further facilitate women's healing through housing, services, and community. Attaining healing is in accordance with the Constitution of the World Health Organization, which specifies that “health is a state of complete physical, mental and social well-being…” (10), and the Universal Declaration of Human Rights, which supports quality housing as a human right (11).

## 2 Trauma

The Substance Abuse and Mental Health Services Administration (SAMHSA) defines trauma as events experienced as physically or emotionally harmful or life-threatening and that negatively affect emotional, physical, social or spiritual well-being (12, 13). The harmful effects are furthermore recognized by SAMHSA as potentially long-lasting. SAMHSA's description is appropriately inclusive in terms of events deemed “trauma”, thus accommodating women's experience of homelessness as well as the harms concurrent with and precipitating homelessness (8, 14), including sexism, ageism and racism.

### 2.1 Trauma of gender violence

Trauma experienced by women has principally been defined and reported in terms of gender-based or intimate-partner violence (IPV) that is physical, sexual, or psychological in nature (15, 16). It is important to highlight gender violence because it is the most common form of violence against women worldwide (15), is a precipitating factor in homelessness (17), and is frequently experienced by women when homeless (18) and at higher rates than among other women (19, 20). Further, Black women experience IPV at significantly higher rates than white women (21, 22) and often with more devastating consequences (23).

There is still relatively limited understanding of the specific experiences and needs of older women who have survived IPV (24). This has been attributed to reporting gaps possibly due to women's accounts being viewed by authorities as less credible because of ageism and sexism (i.e., older women are “unattractive” targets) (24–27) and because of endemic racism against women of color (23, 28). Violence, whether perpetrated by a partner or others, has a significant negative impact on older women's well-being (29). For older women, the consequences of IPV are compounded by the health and functional limitations of “normal” aging (6, 30, 31) and the accelerated aging associated with homelessness (6, 32, 33).

### 2.2 Trauma of intersectional social identities

Trauma is multifold and not limited to gender violence. Older women experiencing homelessness may have multiple social identities for which they are marginalized. Intersectionality refers to possessing or identifying with two or more characteristics that society finds undesirable or unworthy (29, 34, 35). Being a woman, an older person, a Person of Color, and a “homeless person”, for example, subject one to the traumas of racism, ageism, sexism, and “homeism”, where “homeism” refers to discrimination, stigmatization, and prejudice one experiences due to homelessness (36, 37), thus compounding the harms and complicating the pathway to healing.

One manifestation of racism in the U.S. is that Persons of Color are over-represented among those experiencing homelessness (38) due to historical and persistent racial inequities in housing access and wealth-building, among other discriminatory practices. Since the 1980s, these historical racial inequities in housing and wealth have been exacerbated by gentrification, where displacement pressure on low-income individuals and families has had greater and more negative impacts within communities of color (39, 40).

For renters especially, housing security is intimately connected to one's income and financial resources. The percentage of renter households headed by a person of color stood at 48% in 2019, with roughly 58% of Black households living in rental units compared to 28% of white households, and with a high fraction of Black renter households in the lowest income strata (41). Racial discrimination by landlords is widespread, with people of color paying more for identical housing units in the same neighborhoods (42). For 2020–2021, the average earnings of Black women in their 40s was 81% that of White women in the same age range (43).

Women historically have accumulated less wealth relative to men for a variety of reasons including lack of pay equity (44) and time spent outside the workforce in caregiving (45). Many older women living on their own are therefore significantly disadvantaged in monthly Social Security income having earned less over their lifetime, a situation even more acute for older women of color due to the combined effects of gender-based and race-based pay inequities over their working years (46).

## 3 Evidence-based pathways to healing

Amid the harm from myriad traumas experienced over the life course, scholars have sought to understand how older women experiencing homelessness have navigated housing instability and homelessness. Gonyea and Melekis (8) noted enduring challenges for older, homeless-experienced women in constructing a “valued life and self” (8 p68); that is, in healing from traumas. I discuss two essential and foundational elements to healing.

### 3.1 Permanent supportive housing (PSH)

Among evidence-based pathways to healing for older women experiencing homelessness, subsidized PSH – as opposed to temporary shelters or housing where stay is time-limited – is a necessity. For women, this housing is preferably in the form of a private apartment (33). PSH consistent with the Housing First (HF) model (47) is an evidence-based solution to homelessness demonstrated through numerous clinical trials to be effective in remedying homelessness (48, 49). The HF model embodies the principle that all people have a right to adequate housing without prerequisites on accepting services. PSH in this case affords a decent place to live, an environment focused on safety from violence (18), and promotion of well-being through a range of health and supportive services (on-site or nearby) tailored to individual needs and preferences; thus, PSH provides a foundation for healing.

Although PSH is likely the best pathway out of homelessness for older adults including women, the older one is the less likely one is to be placed into PSH (50). PSH facilities and services have thus far prioritized younger people over older persons (37). The disparity in PSH entry by age may reflect insufficiency in services and other necessary accommodations for older adults. Housing solutions must adapt to the changing needs of women as they age to avoid the significant disruption of additional moves and to facilitate rebuilding of “home” (51 p5). Older women should have the right to choose where they reside and to be supported in their chosen home as they age (52). Another challenge is that Black and Hispanic older adults experiencing homelessness have been connected to permanent housing solutions at rates lower than white older adults (50), suggesting not only ageism but racism in policy and practice. Staffing and supportive services that are appropriate and sufficient for addressing the interests and needs of all residents are necessary (53).

There is not an over-abundance of research on outcomes specifically for women in PSH (54), but results from a multi-site clinical trial indicate that women in PSH are more likely to maintain stable residency compared to women in a treatment-as-usual control group (49). An important research finding is that women-only housing options and programs are preferred by women due to trauma experiences (55) but availability is limited. Safe, secure, non-time limited housing is fundamental for older women (56).

### 3.2 Trauma informed care (TIC)

Trauma Informed Care (12, 57) is a necessity in service provision for persons with lived experiences of homelessness due to the prevalence and impact of trauma (14, 31). Stable housing, even PSH, is not sufficient for healing. TIC is a patient-centered, strengths-based, and non-judgmental approach to engaging with and providing services to individuals who have trauma histories (58). TIC was developed in response to the prevalence of trauma and its pervasive and harmful effects, as well as reports of coercive and re-traumatizing practices occurring within health care and other service systems (59).

TIC is furthermore an anti-oppressive practice that acknowledges the harms of racism, sexism, and other forms of oppression (60, 61) so that affected individuals can benefit from services and avoid re-traumatization (59, 62). Within trauma-informed organizations and systems, women have developed a greater sense of empowerment, trust and belief in themselves, and the ability to dream and feel hopeful for their futures (63).

For older women who have endured homelessness, TIC is a best practice (14). A commitment to a trauma-informed workforce, where all staff and service providers are trained, is essential in creating and sustaining an environment of social and emotional safety for trauma survivors (64). A TIC system provides an environment in which trauma survivors can “rebuild a sense of control and empowerment” through a range of programming and supportive services up to and including trauma-focused treatment (12, 65). In the context of serving older women in PSH, then, systemic implementation of TIC is central to healing.

Extending trauma-informed principles to the physical aspects of building interiors further supports PSH residents with comfortable and approachable spaces that facilitate privacy and personal space, for example (66, 67). Providing safe and welcoming common areas also fosters healing by encouraging collaboration and community (68–70).

## 4 Future directions

Several additional pathways to healing hold promise and deserve further attention from researchers and providers in terms of their benefits for older women. I view these additional approaches as augmenting and improving the foundational approaches of PSH and TIC discussed above. These promising pathways would be offered to women within a trauma-informed framework, should they be interested and choose to participate.

### 4.1 Therapeutic recreation (TR)

Introducing TR to the suite of services offered to older women in PSH warrants further assessment and possible widespread adoption. A recent study (71) introduced TR programming into PSH for older adults. Informed by prior studies of TR for people with experiences of homelessness (72), the program offered residents the opportunity to engage in co-designing and participating in their chosen individualized and/or group TR activities over a 30-month period. TR activities fostered an environment where residents developed new friendships and feelings of belonging (71, 73), took on peer-support roles, developed new skills, and began pursuing interests in the community outside the PSH facility (71 p6). Being co-designers of those activities gave residents voice and self-confidence. Overall, the TR activities facilitated transitions from “surviving to thriving” (71 p9). Incorporating co-designed TR programming into PSH as a complement to TIC therefore presents a promising opportunity for older women to realize the inherent value of their lives despite the traumas they have endured.

### 4.2 Mentorship and advocacy

Older women with lived experiences of homelessness and other traumas should be afforded ample opportunities to lend their voices and share their insights and knowledge with others, should they wish to do so. Examples are mentorship and peer navigation, which involve supporting and advising peers on connecting with services and other resources (74). Training and ongoing professional support for peer navigators are necessary since the role is challenging (74, 75). Navigators have described the sense of purpose and fulfillment they derive from helping others (75, 76), thus providing further opportunity for older women to build meaning and value in their lives. It is critical in these contexts that women are appropriately compensated for their time and skills.

Some women may also wish to engage in advocacy [e.g., as in the Advocates Program (77) developed by the Downtown Women's Center in Los Angeles, California], including outreach to local communities and service on advisory boards to lend critical perspectives and expertise to policy makers [e.g., the Lived Experience Advisory Board (78) of the Los Angeles Homeless Services Authority]. Guidance on preparing for public-facing opportunities in light of ongoing stigmatization of homelessness has been developed by the FrameWorks Institute (79).

### 4.3 Reconnection with family

Women with experiences of homelessness often retain emotional ties to family members but typically have only infrequent communications or contact (80). Many women continue to find meaning and value in their past roles as caregivers and express sadness over family ties that have diminished (8), yet research focused on rebuilding older women's social networks is lacking. Helping women re-establish family connections and emotional bonds, including reconciliation with estranged loved ones if this is a woman's choice and is feasible, is another potential pathway to healing for older women. In addition to family members, the opportunity to reconnect with close friends may also be desired and beneficial to healing. Reconciliation efforts with family and friends might be facilitated by on-site staff such as counselors, if women desire such assistance.

## 5 Discussion

PSH and TIC are two key evidence-based pathways through which older women with lived experiences of homelessness can achieve healing; that is, a valued life and self. A number of other highly promising pathways to facilitate healing complement TIC within the PSH setting and deserve further investigation specific to older women and wider implementation. PSH and TIC have proven benefits and therefore should be expanded so that all older women experiencing homelessness have the opportunity to be served and to heal. Social and moral costs of failing to meet the women's needs are unacceptable.

Failing to address the drivers of traumas and the harms they cause is also unacceptable. The highly disproportionate number of Persons of Color experiencing or under threat of homelessness in the United States, relative to their representation in the U.S. population, is the result of systemic and compounded intergenerational inequalities. Persons of Color have been denied equal opportunities for employment and to earn equitable wages, own property, and build wealth among other harms of racism. Women, too, have been harmed by the structural inequalities stemming from sexism, and for older women those inequities have been compounded over the life course and complicated by ageism. Because present inequities are the cumulative result of unjust policies and practices, fundamental changes to policies and practices are necessary to correct the inequities.

A paradigm shift in U.S. homelessness policy is needed. We can look abroad for examples of bold policy initiatives that have been effective. In Wales, legislation was adopted in 2014 that imposed a legal “Duty-to-Assist” obligation on government agencies and authorities to take all reasonable steps to prevent individuals from becoming or remaining homeless (81–84). Emphasis was focused as much on prevention as on offering permanent housing to those already homeless. In the first year after the “Duty-to-Assist” policy became law, the number of people in Wales experiencing homelessness was reduced by roughly 2/3rds (85, p12). In Canada, Van Berkum and colleagues (86) outline a systems-approach to address homelessness among older adults, ranging from income support and benefits aimed at preventing loss of housing to a comprehensive “Duty to Assist” policy to ensure those currently experiencing homelessness receive PSH accompanied by age-appropriate services as needed.

In the U.S., one example of what focused efforts can accomplish is the “Ending Veteran's Homelessness” initiative launched by the Dept. of Veterans Affairs that embraced a Housing First approach and reduced the number of homeless veterans by more than 55% from 2010 to 2024 (87). A nationwide “Duty-to-Assist” policy in the U.S., whether focused only on older adults or all persons experiencing homelessness, would require governmental agencies and authorities to use all resources at their disposal such as vouchers, housing subsidies, payments of overdue rent, and other measures – regardless of cost – to prevent eviction and assist those currently experiencing homelessness to transition into safe, stable, permanent supportive housing. Success would also require addressing the widespread shortage of available housing stock suitable for PSH (88, 89).

Gender violence continues to be a destabilizing and profoundly harmful force in women's lives. Like the policies needed to proactively prevent and eliminate homelessness, there must be a similar “Duty to Assist” commitment enacted to address gender violence. The U.S. Violence Against Women Act, Reauthorization Act of 2022 (90) mandates shelter assistance and contains some protections against evictions due to domestic violence, but this is insufficient and does not specifically address older women experiencing homelessness. Although the United Nations Sustainable Development Goals call for the elimination of gender violence, there has been minimal progress toward this goal (91).

In closing, we must continue striving to realize the humanitarian goals of quality housing (11) and complete well-being (10) as human rights, as agreed upon by nations of the world.

## Data Availability

The original contributions presented in the study are included in the article/Supplementary Material, further inquiries can be directed to the corresponding author.
